# Family function and anxiety among junior school students during the COVID-19 pandemic: a moderated mediation model

**DOI:** 10.3389/fpsyt.2023.1217709

**Published:** 2023-06-22

**Authors:** Zhifang Guo, Juan Zhao, Jiani Peng

**Affiliations:** School of Education Science, Shangrao Normal University, Shangrao, China

**Keywords:** anxiety, family function, perceived stress, junior school students, COVID-19 pandemic

## Abstract

**Background:**

The prevalence of anxiety among adolescents is relatively high during an epidemic. Studies have reported that family function and perceived stress are important factors affecting adolescents’ anxiety. However, only few studies have explored the factors influencing the relationship between family function and anxiety. Therefore, this study explored the mediating and moderating mechanisms underlying this relationship among junior school student during the COVID-19 pandemic.

**Methods:**

745 junior school students completed family function, perceived stress, and anxiety questionnaires.

**Results:**

(1) The junior school students that were left-behind tended to show lower family function (*t* = −4.21, *p* < 0.001), greater perceived stress (*t* = 2.72, *p* < 0.01), and higher anxiety (*t* = 4.24, *p* < 0.001), (2) Family function in junior school students was negatively associated with anxiety (*r* = −0.35, *p* < 0.001); perceived stress mediated the relationship between family function and anxiety (*p* < 0.001), and (3) Whether the student was left-behind (LB) moderated the link between family function and anxiety (*β* = −0.16, *t* = −3.33, *p* < 0.001) and between family function and perceived stress (*β* = −0.22, *t* = −2.61, *p* < 0.001).

**Conclusion:**

These findings suggest a negative association between family function and anxiety. Knowledge of the mediating role of perceived stress and moderating role of being left-behind may help prevent and improve anxiety among junior school students during the COVID-19 pandemic.

## 1. Introduction

In recent years, the pandemic has had a significant impact on the psychological health of adolescent students, the most prominent manifestation of which is that more students are experiencing anxiety symptoms (1–3). A study showed that 54% of adolescent students in China consider their learning and graduation to be affected by the COVID-19 pandemic and have significant anxiety (4). Panda et al. (5) used a meta-analysis to analyze abnormal psychological behaviors among children, adolescents, and caregivers, and the results showed that the incidence of anxiety symptoms during the pandemic period reached 34.5%, particularly among adolescent students. During the epidemic, the level of anxiety among adolescents is relatively high due to various factors. In addition to the stress caused by studying and graduating during the pandemic, the impact of factors such as the family environment and imbalances in physical and psychological development caused additional stress and also brought many anxieties to adolescents, especially junior school students (6–9). Anxiety is a risk factor for negative behaviors such as violence, addictive behavior, and eating disorders (10). It therefore increases the risk of self-injury and suicide among adolescents (11). Therefore, individual anxiety in junior school students has attracted increasing attention from researchers.

### 1.1. Family function and anxiety

Ecosystem theory points out that the family growth environment is an indispensable factor that has a significant impact on the healthy growth of children. Family function is an important indicator of the functioning of the family system and is a deep-seated variable that affects the psychological development of family members. Studying the factors that influence family function has important theoretical value and practical significance (12). According to Olson’s circular pattern theory, family function is the emotional connection between family members, rules within the family, communication, and interaction between members, and the effectiveness of coping with emergencies. The effectiveness of family function is often related to intimacy and adaptability (13). Studies have shown that family function is significantly correlated with adolescents’ emotional health and problematic behavior (14). Adolescents with good family function are less likely to have psychological problems, indicating that family function can significantly and positively predict an individual’s mental health level (15). The more complete the family functions, the lower the social anxiety among children and adolescents; incomplete family functions are positively correlated with anxiety (16, 17). Family function plays an important role in influencing individual anxiety, and excessive parental control and lack of emotional expression can lead to high anxiety in adolescents (18).

### 1.2. Perceived stress as a mediator

Negative thoughts and feelings can generate when an individual experiences great stress in response to stressful life events. Negative thoughts and feelings are known as perceived stress (19). The cognitive theory of stress states that cognitive evaluation is an important factor in individual responses to stress (20), and the results of perceived stress are different due to their cognitive evaluation of stress; when an individual is faced with stress, the effectiveness of stress factors depends on the individual’s perceived stress (21). Perceived stress exacerbates negative physical and physiological outcomes (22). Previous studies have shown that family communication between adolescents and children is closely associated with perceived stress (23). Effective family communication can encourage individuals to actively respond to stressful situations and decrease perceived stress (24, 25). The theory of social ecology emphasizes that, when compared to objective social situations, individuals’ perceived social psychological situations are more closely related to individual reactions (26). Research has shown that individuals’ cognitive levels of stressful events affect their emotional state, and that individuals who perceive more stress are more likely to generate negative emotions (27). The cognitive model of anxiety also indicates that its mechanism is mainly the result of the continuous development of beliefs characterized by a lower sense of control over the environment (28). Some studies have pointed out that the greater the perceived stress, the easier it is for individuals to experience a sense of tension and loss of control, and therefore have higher their anxiety levels (29).

Accordingly, the following research assumptions are proposed:

*Hypothesis 1*. Perceived stress mediates the relationship between family function and anxiety in junior school students.

### 1.3. Left-behind status as a moderator

The mediating model, which examined the relationship between family function and anxiety among junior school students, has important theoretical significance in explaining the internal psychological significance and impact of external events. At the same time, the impact of family function on perceived stress and anxiety may be regulated by other factors, which can better explain the “conditions” of influence among various variables (30). This not only promotes the comparability of research in the research fields of anxiety but also provide ideas for preventing and intervening in negative impacts on family functions concerning anxiety among junior school students in order to find more effective prevention and intervention measures.

Junior school students who are left-behind refer to those who stay at home because their parents or single parents have worked outside for a long time. They are often classified as left-behind children in a broad sense (31). Left-behind children generally refer to children under the age of 18 whose one or both parents emigrated or worked abroad or worked at home or abroad for more than 3 months, and were left in the place of registered residence (32). Research has shown that the disadvantageous situation of parent–child separation makes left-behind junior school students more likely to perceive external pressure (33). At the same time, in the case of parent–child separation, the family functions of left-behind children are negatively affected to varying degrees, which has a negative impact on their mental health; thus, left-behind children are prone to various psychological problems (34–36). Some studies have pointed out that compared to non-left-behind junior school students, left-behind junior school students generally have poorer family functions and more problematic behaviors (29, 37). During the pandemic, left-behind children may not be with accompanying parents at home, however, even with accompanying parents, the family function of left-behind children has not been improved perhaps due to factors such as the quality of parental participation and the formation of parental attachment (38). There is significant negative correlation between family function and mental health, e.g., anxiety among left-behind high school students (39). Some studies have shown that a lack of family function in left-behind children can lead to more negative emotional experiences, which can lead to depression and anxiety (40). Compare to non-left-behind adolescents, the effect of family function on prosocial behavior through peer acceptance was stronger than those former/present left-behind adolescents (41), and emotions themselves are behavioral dynamics (42), those can be considered that it is inherently related to behavior. Thus, this study proposes the following hypotheses:

*Hypothesis 2*: Being left-behind by parents moderates the relationship between family function and anxiety among junior school students.

Family function is a part of the family environment (43). The lifelong development model of the relationship between stress and health indicates that adverse family environments such as apathy or neglect experienced by individuals in their early stages can reduce their psychological resources, such as positive beliefs related to optimism (44), and the positive beliefs associated with optimism leads to a lesser response to stress, that is, stress events are less likely to be perceived by individuals (45). According to the model of “delivering charcoal in the snow” proposed by Li Dongping (46), this model believes that individual risk factors play a moderating role in the relationship between ecological resource factors and social adaptation. The developmental disadvantage of individuals with high individual risk is reflected more in the situation of low ecological resources rather than high ecological resources, compared to those with low individual risk. According to this model, compared to junior school students who were not left-behind, left-behind junior school students experienced a faster decrease in stress perception as their family functions improved. Thus, we propose the following hypotheses:

*Hypothesis 3*: Being left-behind by parents moderates the relationship between family function and perceived stress among junior school students.

**Figure 1:**
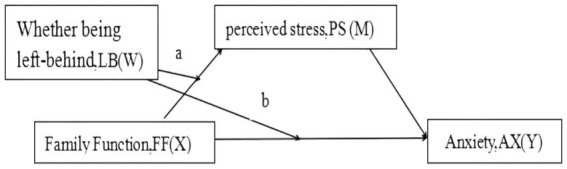
(Hypothesis model): the moderated mediation model.

Based on these theories and research assumptions, this study proposes the following mediated model: (Figure 1). Specifically, family function of junior school students not only directly predicts anxiety, but also affects anxiety levels through perceived stress, i.e., there is an impact path of family function→ perceived stress→ anxiety. At the same time, left-behind status plays a moderating role between family function and anxiety as well as between family function and perceived stress.

## 2. Measures

### 2.1. Participants

A total of 745 participants (55.17% female) were included in the analysis. The sample was composed of first-(33.56%), second-(33.42%), and third year (33.02%) students; 41.61% of the participants were junior school students who were left-behind.

### 2.2. Family function

The family function scale consisted of 20 items (e.g., “Respect friends of other family members”). It was originally developed by Olson et al. in 1985 (13) to evaluate individuals’ levels of family function, and the Chinese version was revised in the context of Chinese culture by Xu Jie, Fang Xiaoyi, et al. (47) to include two dimensions: family affinity and adaptability. All responses were measured on a 5-point Likert scale (1 = strongly disagree to 5 = strongly agree). In the present study, the reliability coefficient of the scale was 0.88.

### 2.3. Perceived stress

The Chinese version (48) of the perceived stress Scale (49) was used to measure participants’ levels of perceived stress, it consisted of 14 items (e.g., “Feel nervous and stressed”). Individuals rated each item on a 5-point Likert scale (1 = Never,5 = Always), α = 0.85.

### 2.4. Anxiety

The Self-rating anxiety scale (50) was initially developed by Zung in 1971 for evaluating anxiety. The Chinese version (51) was revised by Wang et al. The scale consisted of 20 items (e.g., “easy to be upset”) and all items were measured on a 4-point Likert scale (1 = Never, 4 = Always). In this study, the reliability coefficient of the scale was 0.86.

## 3. Data analysis

SPSS23.0 was used to analyze the data. Pearson’s correlation coefficient was used for descriptive statistics. The PROCESS Models 4 and 8 macros for SPSS were used to test the mediation and moderated mediation models, respectively. Indirect and direct effects were estimated using 5,000 bootstrap samples. Significance was evaluated using 95% confidence intervals (CIs). All variables were standardized prior to analysis.

## 4. Results

### 4.1. Preliminary analysis

The means, SDs and Pearson correlations among the variables are presented in Table 1. There was a significant negative correlation between family function and perceived stress (*r* = −0.21, *p* < 0.001). Family function was negatively correlated with anxiety (*r* = −0.35, *p* < 0.001). Perceived stress was positively correlated with anxiety (*r* = 0.34, *p* < 0.001).

**Table 1:** Descriptive statistics and correlations.

Variables	*M*	*SD*	1	2	3
1 perceived stress	2.66	0.83	1		
2 Family function	3.01	0.71	−0.21^***^	1	
3 Anxiety	2.09	0.51	0.34^***^	−0.35^***^	1

*N* = 745; M, mean; SD, standard deviation; ^*^p < 0.05, ^**^p < 0.01, ^***^p < 0.001.

### 4.2. Comparison of the three variables

The results showed there was a significant difference in the three variables of perceived stress, family function, and anxiety between junior school students who were left-behind and those not left-behind. Left-behind junior school students tended to show higher anxiety than the others (*t* = 4.24, *p* < 0.001). The family function level of junior high school students who were left-behind was significantly lower than that of junior high school students who were not left-behind (*t* = −4.21, *p* < 0.001). Junior school students who were left-behind tended to show greater perceived stress than the others (*t* = 2.72, *p* < 0.01).

### 4.3. Analysis of perceived stress as a mediator

As shown in Table 2, Equation 1 (anxiety), family function was negatively related to anxiety (*β* = −0.25, *t* = −10.01, *p* < 0.001). According to Equation 2 (perceived stress) and Equation 3 (anxiety), family function was significant negatively related to perceived stress (*β* = −0.24, *t* = −5.85, *p* < 0.001) and significant negatively related to anxiety (*β* = −0.21, *t* = −8.48, *p* < 0.001), and perceived stress was significant positively related to anxiety (*β* = 0.17, *t* = 8.22, *p* < 0.001). Hypothesis 1 was verified; that is, perceived stress mediates the relationship between family function and anxiety.

**Table 2:** The mediation model.

Predictors	Equation 1	Equation 2	Equation 3	Equation 4	Equation 5
	(Anxiety)	(Perceived stress)	(Anxiety)	(Perceived stress)	(Anxiety)
	*β* (95% CI)	*β* (95% CI)	*β* (95% CI)	*β* (95% CI)	*β* (95% CI)
Grade	0.01 (−0.03, 0.005)	0.05 (−0.01, 0.12)	0.01 (−0.04, 0.04)	0.06 (−0.01, 0.13)	0.01 (−0.03, 0.04)
Gender	0.04 (−0.03, 0.11)	0.01 (−0.11, 0.12)	0.04 (−0.02, 0.11)	0.02 (−0.09, 0.13)	0.05 (−0.01, 0.11)
Family function	−0.25^***^ (−0.29, −0.19)	−0.24^***^ (−0.32, −0.16)	−0.21^***^ (−0.25, −0.15)	−0.22^***^ (−0.30, −0.14)	−0.19^***^ (−0.23, −0.14)
perceived stress			0.17^***^ (0.13, 0.21)		0.16^***^ (0.12, 0.20)
left-behind status				0.12^***^ (−0.01, −0.23)	0.09^***^ (0.02, 0.16)
Family function* left-behind status				−0.22^***^ (−0.38, −0.05)	−0.16^***^ (−0.25, −0.07)
*R^2^*	0.12	0.05	0.19	0.06	0.21
*F*	34.29^***^	12.05^***^	44.95^***^	9.52^***^	33.79^***^

Each equation controls the grouping of grade and gender; ^*^p < 0.05, ^**^p < 0.01, ^***^p < 0.001.

### 4.4. Analysis of left-behind status as a moderator

In Table 2, Equation 4 (perceived stress) examined the moderation effect of left-behind status on path a (Figure 1) (*β* = −0.22, *t* = −2.61, *p* < 0.001), while Equation 5 (anxiety) examined the moderation effect of left-behind status on path b (Figure 1) (*β* = −0.16, *t* = −3.33, *p* < 0.001).

**Figure 2:**
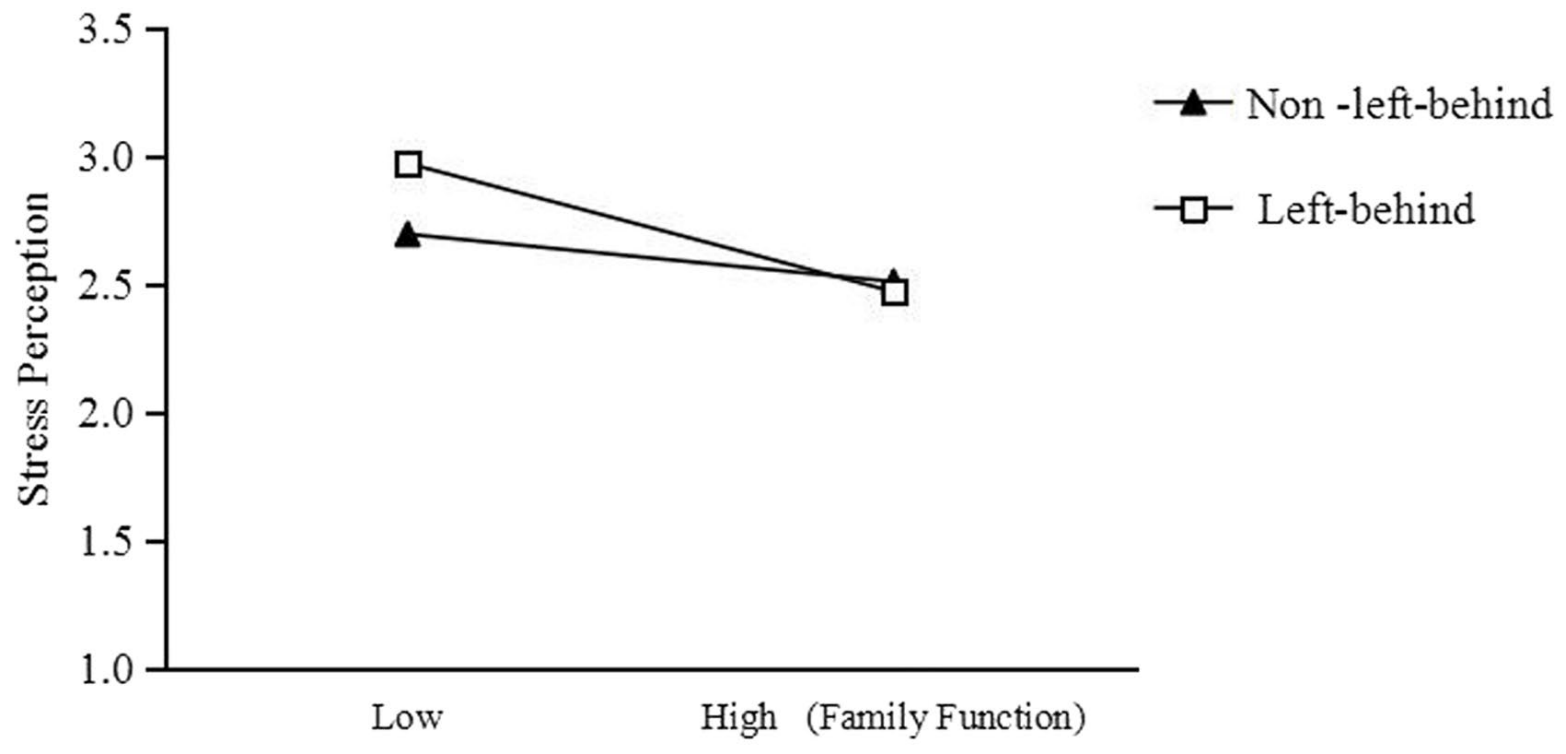
Interaction between family function and whether being left-behind on perceived stress.

**Figure 3:**
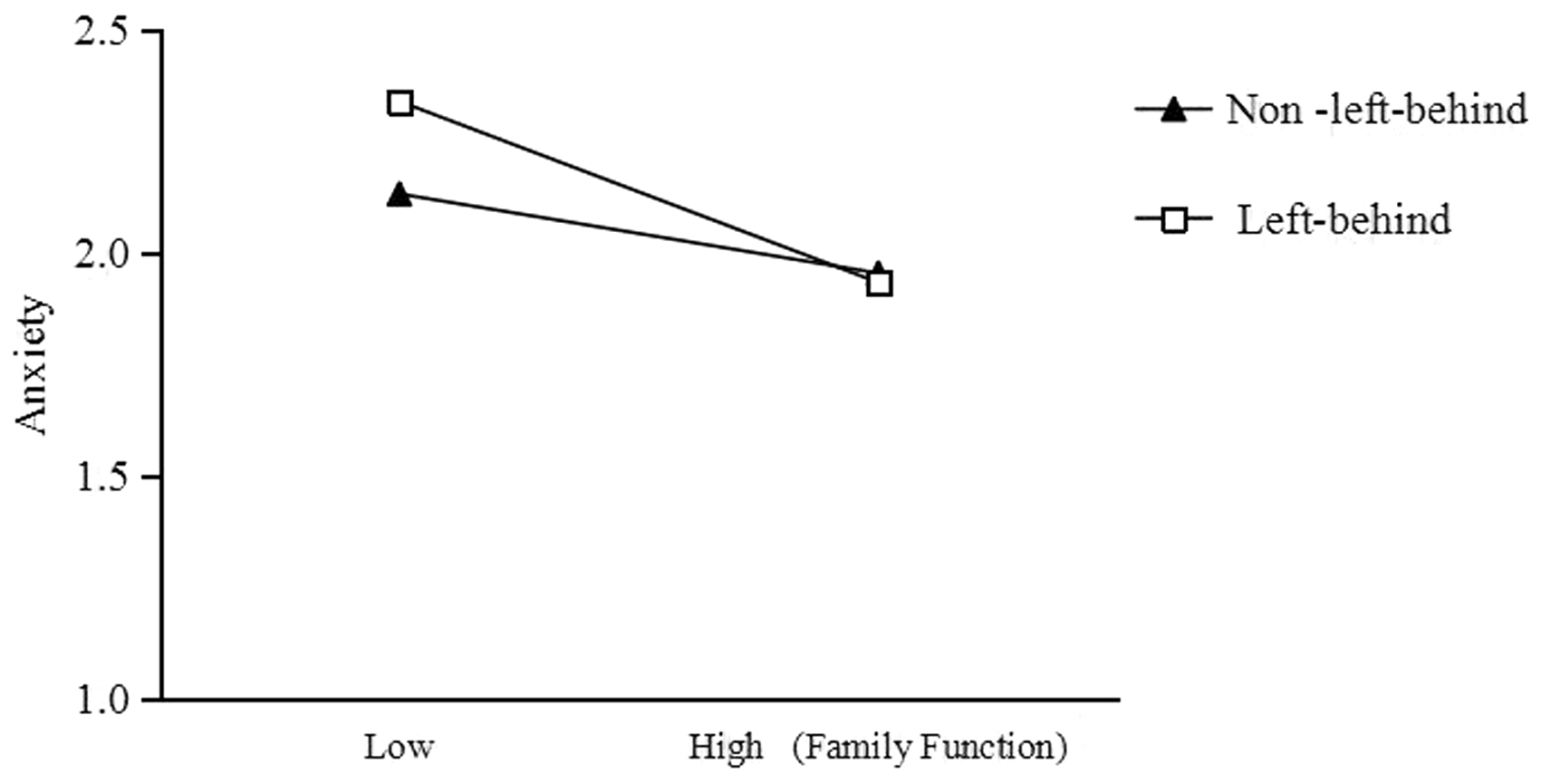
Interaction between family function and whether being left-behind on anxiety.

Simple slopes were probed to further explore the moderating role of being left-behind in the mediation association (Figures 2, 3). Family function had a significant negative effect on perceived stress as well as on anxiety between junior school students who were left-behind and those not left-behind. The effect of family function on perceived stress was stronger for junior school students who are left-behind (*b*_simple_ = −0.35, *t* = −5.66, *p* < 0.001) than for others (*b*_simple_ = −0.13, *t* = −2.25, *p* < 0.05). The effect of family function on anxiety was stronger for left-behind junior school students (*b*_simple_ = −0.28, *t* = −7.96, *p* < 0.001) than for others (*b*_simple_ = −0.13, *t* = −3.82, *p* < 0.001). Thus, Hypotheses 2 and 3 were verified.

## 5. Discussion

### 5.1. Comparison of differences among three variables

The family function of junior school students who are left-behind was significantly lower than that of junior school students who were not left-behind, and their stress perception was significantly higher than that of students who were not left -behind. Compared to these children, the frequency and quality of communication between children who are left-behind and their parents is relatively low (52), leading to emotional apathy among family members, and these students were worried about their learning and living status, therefore were prone to high stress perception, which was precisely due to poor family function.

This study showed that junior school students who were left-behind tended to report greater anxiety than their counterparts, which was consistent with previous studies (53, 54). On the one hand, due to insufficient education and guidance for junior school students who were left-behind, they became introverted and sensitive, were not good at or afraid of communicating with others, and harbored enmity toward others. They were prone to emotional anxiety, nervousness, and difficulty in calming down (55). On the other hand, owing to the impact of pandemic prevention and control, some junior school students who were left-behind lacked the correct guidance of their parents for various types of pandemic information because their parents were unable to stay home. Consequently, panic and anxiety were exacerbated because they were unable to distinguish the authenticity of the information and accurately assess the risk information of the epidemic. As can be seen that the disadvantaged situation of left-behind junior school students still deserves academic attention.

### 5.2. The mediating effect of perceived stress

The mediating effect indicated that perceived stress was an important bridge between family function and anxiety. Perceived stress was proven to be related to family function. During COVID-19, influenced by risk information, ability to get along with family members, sudden changes in learning styles, social isolation, and other factors influenced junior school students were prone to falling into certain stressful situations, and their mental stress increased significantly (56). In the same stressful situation, some students experienced high levels of stress, while others experienced low levels of stress. That is, compared to students with poor family function, junior school students with good family function might effectively reduce their perceived stress.

The results showed that perceived stress was positively correlated with anxiety, indicating that when junior school students had higher levels of perceived stress, their anxiety levels were also higher, whereas when they had lower levels of perceived stress, their anxiety levels were also lower. This result was consistent with those of previous research results (57–59).

According to the Cognitive Phenomenon Logical Transaction (CPLT) model of stress, the stress response mainly depends on an individual’s understanding of the overall relationship between themselves and their environment (20). Therefore, families should not excessively increase their children’s academic burden, and provide them with appropriate leisure time to reduce their stress perception (60). Meanwhile, effective measures, such as improving the family environment of junior school students, should be taken to reduce psychological stress caused by the pandemic, effectively improve family cohesion, and better exert family functions. It was also possible to provide psychological intervention by improving their mental adjustment and coping methods, leading them to form positive values, and helping them positively face stressful situations to reduce negative emotions such as anxiety.

### 5.3. The moderating effect of left-behind status

Compared to other junior school students, left-behind junior school students’ family function was strongly negatively correlated with perceived stress. According to the “delivering charcoal in the snow” model (46) and compared to those with lower individual risks, the disadvantage of individuals with higher individual risks was in the situation of low ecological resources and not in high ecological resources. Therefore, left-behind junior school students exhibited higher stress perception only when their family function was low. Owing to the long-term absence of left-behind junior school students, parents can regularly communicate and exchange ideas through the Internet. Once problems are discovered, parents can quickly find appropriate opportunities to provide creative guidance, helping them view setbacks, gains, and losses with the correct mindset, making them truly realize that their parents are always their strongest source of support, and encouraging them to actively face their lives. Maintaining and strengthening parent–child communication can alleviate the various stresses perceived by children by their parents (60).

Compared to other junior school students, the family function of junior school students who are left-behind was negatively correlated with anxiety. This may be because children who are left-behind have significantly lower family attention than those not left-behind (61), and they crave love and support from their families more urgently. They are eager to receive more emotional communication and understanding between parents (62) to compensate for the negative effects of parent–child separation on children’s growth (63). For junior school students who are left-behind with poor family functions, family therapy can be used to reduce their perceived stress and improve their negative emotions; that is, based on the individual situation of junior school students, starting from their past negative experiences, it can be helpful to alleviate their anxiety by changing their cognition and experience of negative family events that occurred in the past, venting their emotions, and rebuilding more adaptive interaction patterns among family members (64), meanwhile, all circles should carry out the lectures of family education, spread scientific educational concepts and help caregivers to change their unscientific family concepts and child views (65).

### 5.4. Research implications and limitations

This study explored the relationship between family function and anxiety among junior school students. It not only helps us understand how perceived stress affects anxiety among junior school students but also demonstrates the importance of family function in individual growth. Establishing and maintaining a harmonious parent–child relationship, creating a reasonable family atmosphere, and making the family function play a good role is crucial for the happy growth of junior school students. Through incremental care and emotional support from their parents, children can experience warmth and care for their families. Meanwhile, to improve the family atmosphere and promote good functioning of the family, parents should communicate effectively with their children and establish harmonious parent–child relationships. This is crucial for the growth of junior school students. The moderating role indicated that junior school students who were left-behind need their parents to handle the relationship between material and spiritual support and adopt various channels to strengthen family functions to improve their negative emotions.

This study had certain limitations. First, it only explored the impact mechanism of perceived stress on junior school students’ anxiety through cross-sectional research. In the future, longitudinal research will be combined to better clarify the relationship between various variables. Second, our research data were collected in a continuous epidemic situation, reflecting the relationship between variables of the COVID-19 epidemic. With the changes in COVID-19, the relationship between the above variables is tenable remains to be verified.

## 6. Conclusion

The present study found that direct and indirect relationships between family function and anxiety, as well as left-behind status, simultaneously moderated the mediating effect. To prevent and reduce anxiety among junior school students, improving family function may be coupled with reducing stress perception levels to further mitigate stress onset. When considering the prevention and improvement of anxiety among junior school students who were left-behind, a beneficial approach is to improve family function.

## Data availability statement

The original contributions presented in the study are included in the article/supplementary material, further inquiries can be directed to the corresponding author.

## Ethics statement

The studies involving human participants were reviewed and approved by Research Ethics Committee of Shangrao Normal University. Written informed consent to participate in this study was provided by the participants’ legal guardian/next of kin.

## Author contributions

ZG contributed to the design of the research drafting the manuscript. JZ contributed to the revision of the manuscript. JP contributed to the arranging of materials and data. All authors contributed to the article and approved the submitted version.

## Funding

This research was supported by “Research on Academic Emotion of Children from Difficult Families,” a key project of the Ministry of Education in the 13th Five Year Plan of National Education and Science of China (DBA190310).

## Conflict of interest

The authors declare that the research was conducted in the absence of any commercial or financial relationships that could be construed as a potential conflict of interest.

## Publisher’s note

All claims expressed in this article are solely those of the authors and do not necessarily represent those of their affiliated organizations, or those of the publisher, the editors and the reviewers. Any product that may be evaluated in this article, or claim that may be made by its manufacturer, is not guaranteed or endorsed by the publisher.
